# Combining classifiers to predict gene function in Arabidopsis thaliana using large-scale gene expression measurements

**DOI:** 10.1186/1471-2105-8-358

**Published:** 2007-09-21

**Authors:** Hui Lan, Rachel Carson, Nicholas J Provart, Anthony J Bonner

**Affiliations:** 1 Department of Computer Science, University of Toronto, 40 St George St, Toronto, ON M5S 2E4, Canada; 2 Department of Cell and Systems Biology/Centre for the Analysis of Genome Evolution and Function, University of Toronto, 25 Wilcocks St, Toronto, ON M5S 3B2, Canada

## Abstract

**Background:**

*Arabidopsis thaliana *is the model species of current plant genomic research with a genome size of 125 Mb and approximately 28,000 genes. The function of half of these genes is currently unknown. The purpose of this study is to infer gene function in Arabidopsis using machine-learning algorithms applied to large-scale gene expression data sets, with the goal of identifying genes that are potentially involved in plant response to abiotic stress.

**Results:**

Using in house and publicly available data, we assembled a large set of gene expression measurements for *A. thaliana*. Using those genes of known function, we first evaluated and compared the ability of basic machine-learning algorithms to predict which genes respond to stress. Predictive accuracy was measured using ROC_50 _and precision curves derived through cross validation. To improve accuracy, we developed a method for combining these classifiers using a weighted-voting scheme. The combined classifier was then trained on genes of known function and applied to genes of unknown function, identifying genes that potentially respond to stress. Visual evidence corroborating the predictions was obtained using electronic Northern analysis. Three of the predicted genes were chosen for biological validation. Gene knockout experiments confirmed that all three are involved in a variety of stress responses. The biological analysis of one of these genes (At1g16850) is presented here, where it is shown to be necessary for the normal response to temperature and NaCl.

**Conclusion:**

Supervised learning methods applied to large-scale gene expression measurements can be used to predict gene function. However, the ability of basic learning methods to predict stress response varies widely and depends heavily on how much dimensionality reduction is used. Our method of combining classifiers can improve the accuracy of such predictions – in this case, predictions of genes involved in stress response in plants – and it effectively chooses the appropriate amount of dimensionality reduction automatically. The method provides a useful means of identifying genes in *A. thaliana *that potentially respond to stress, and we expect it would be useful in other organisms and for other gene functions.

## Background

Assigning functions to unannotated genes, identified by genome sequencing and other methods, is the goal of functional genomics. Many approaches have been proposed for large-scale prediction of gene function [1-6]. These approaches are largely based on physical association, genetic interaction, sequence relationships and patterns of gene expression. Predicting gene functions based on large-scale gene expression measurements is an attractive strategy since many pathways display coordinated transcriptional regulation [2,7]. Although previous studies show that supervised learning methods can be used to predict gene function based on gene expression in microorganisms such as the yeast *Saccharomyces cerevisiae *and in mammals such as mice [1,8-16], it remains unknown to what extent this is true in plants.

With the *A. thaliana *genome completely sequenced [17], functional annotation of the genes remains a key challenge for biologists. Currently, approximately 50% of the 28,000 genes have not been assigned any function [18]. Thus, the extent to which supervised learning methods can be used to infer gene function in *A. thaliana *is a timely and important question. Little work has been done in this area, two exceptions being [19,20].

In [19], a method is developed to infer gene function from microarray data and predicted protein-protein interactions. The method is similar to Nearest Neighbor algorithms [21] in that the predicted function(s) of a gene are based on the function(s) of nearby genes. Here, the "nearness" of one gene to another is based on a normalized Pearson correlation of their expression profiles and on putative interactions of their protein products. In addition, the method is extended to the discovery of biological pathways, and is applied to predicting the signaling pathway of phosphatidic acid as a second messenger in *A. thaliana*.

In [20], a decision tree algorithm is applied to the problem of predicting the function of protein sequences in *A. thaliana*. Six sources of data were used: sequence, expression, SCOP, secondary structure, InterPro and sequence similarity. One conclusion of the study is that the decision tree algorithm was unable to extract much information from the expression data. The authors suggest that this is because the expression data came from unrelated and highly-specific experiments with just a few readings per gene each. They also suggest that because many more expression data sets are now available for *A. thaliana*, results may improve when using this type of data in the future.

The present study aims to identify unannotated genes in *A. thaliana *that are potentially involved in plant response to stress. In the context of plants, a stress (biotic or abiotic) causes a decrease in plant growth or yield. We investigated the prediction of gene function in *A. thaliana *based solely on gene expression data using a variety of basic supervised learning methods, namely Logistic Regression (LR), Linear Discriminant Analysis (LDA), Quadratic Discriminant Analysis (QDA), Naive Bayes (NB) and K-Nearest Neighbors (KNN). We also investigated the effect on the learning methods of preprocessing the expression data using Principal Component Analysis (PCA). Finally, we improved the performance of the basic learning methods by combining them using a weighted voting (WV) scheme. This work has enabled our collaborators, biologists in the Department of Cell and Systems Biology at the University of Toronto, to carry out directed biological experiments for determining gene function. In addition to these biological results, the paper illustrates how various machine-learning methods have had to be adapted to fit this bioinformatics application.

## Results and discussion

### Microarray data and the Gene Ontology

In this study, we used two microarray data sets: one from the Botany Array Resource at the University of Toronto [22], and the other from the AtGenExpress Consortium [23], archived at NASCArrays [24,25]. These data sets include over 1000 expression-level experiments for *Arabidopsis*, and using all of them would give a data set with dimensionality over 1000. Since the performance of statistical and machine-learning methods tends to decrease with dimensionality, we chose only those experiments that are specifically stress-related. Even so, the covariance matrix of the resulting data set is singular, which is a problem for many of the machine-learning methods. The singularity is probably due to dependencies between the expression levels under control conditions, since removing the controls from the data sets solved the problem. To compensate, we tried applying the learning algorithms to expression-level ratios (*i.e*., ratios of experimental to control conditions). However, we found that the results were better when ratios were not used (data not shown). This is probably because the classifiers look for genes that respond similarly to the known stress-associated genes, so it is not so important to include the controls. In addition, since many of the features are time-courses, there is still a "time zero" control included for the values, providing a baseline measurement. The results reported in this article are therefore based on absolute expression levels without controls.

From the Toronto data set, we selected 54 features corresponding to experiments conducted primarily to study plant environmental and stress physiology, plant physiology, plant-microbe and plant-insect interactions. From the AtGenExpress data set, we selected 236 features, including various abiotic stresses (e.g., osmotic stress, heat stress, cold stress, salt stress, drought stress, UV-B stress, wounding stress, water-deprivation stress and oxidative stress). We combined the selected features into a single data set. The resulting data set consists of gene expression levels for 22,746 genes under 54 + 236 = 290 different experimental conditions.

We used terms from the Gene Ontology for Biological Processes (GOBP) to represent gene function. For example, the GOBP term *GO:0006950 [response to stress] *refers to genes that respond to stress. In general, the Gene Ontology (GO) provides a dynamic controlled vocabulary for describing genes and gene products in any organism [26]. "Biological Process" is one of three broad GO categories (the other two being "Molecular Function" and "Cellular Component"). GOBP terms are organized into a directed acyclic graph (DAG) to reflect the hierarchical relationships between the terms. Parent GOBP terms are subdivided into increasingly specific child GOBP terms.

Since our study focussed on stress, we were concerned with gene functions at or below the term *GO:0006950 [response to stress] *in the GOBP hierarchy. This GOBP term has 19 child terms, such as *GO:0009409 [response to cold]*, *GO:0009408 [response to heat]*, and *GO:0009414 [response to water deprivation]*. Since gene function becomes more and more specific as we move down the GOBP hierarchy, fewer and fewer genes have any given annotation. The result is that for specific types of stress, our data set contains many negatives and few positives. In the best case, for the term *GO:0009613 [response to pest, pathogen or parasite]*, over 97% of the training data consists of negatives. The typical case is even worse. In fact, looking at all 19 types of stress, 5 types have no positives at all, and of the remaining 14 types, the median number of negatives is 99.2% of the training data. This highly unbalanced data made accurate prediction of gene function difficult. For this reason, we narrowed our study to the top stress term, *GO:0006950 [response to stress]*. To get positive training samples for this term, we propagated all genes in its offspring upward to it in the hierarchy. After up-propagation, the top stress term has 1,031 genes, or almost 9% of the total genes in the training data. The training data therefore contains 9% positives and 91% negatives.

Using GOBP terms to annotate all genes in *A. thaliana *is an ongoing project started in 2002 by TAIR [27,28]. The gene annotations (updated weekly) can be downloaded from TAIR [27]. The predictions reported in this paper are based on the version for March 10, 2007. Using these annotations, we categorized the genes into *annotated *genes and *unannotated *genes. The annotated genes are those which have at least one GOBP annotation; the unannotated genes are those which have no GOBP annotations. In addition, a gene was treated as unannotated if its only annotation is the top GOBP category, *GO:0008150 [biological process]*, since the function of such a gene is unknown. The result was 11,553 annotated genes and 11,193 unannotated genes in our data set.

The annotated genes formed the training data, in which a gene was called positive if it is annotated as a stress gene, and negative otherwise. The unannotated genes formed the prediction data. It should be noted that this approach probably introduces some false negatives into the training data, because genes not known to have a particular function are considered to be negative, even though future experiments could reveal them to have that function. That is to say, "unknown" is treated as "negative". However, the number of such false negatives should be small, since only a small number of genes participate in any given biological process. That is, most negatives are true negatives.

### Predicting gene function using basic learning methods

Using a variety of basic learning methods, we trained a number of classifiers to distinguish between genes that do and do not respond to stress, based on their patterns of gene expression in the training data. We then applied each classifier to the prediction data to estimate the function of the unannotated genes. In addition, we used cross validation to evaluate the performance of each classifier and to estimate the precision of each prediction.

We used five supervised learning methods: Logistic Regression (LR), Linear Discriminant Analysis (LDA), Quadratic Discriminant Analysis (QDA), Naive Bayes (NB) and K-Nearest Neighbors (KNN) [21] (see Methods). These methods were chosen because they are representative of the most basic supervised learning methods, the goal being to explore simple methods first. These methods are widely understood, take little computation time, and the results provide a benchmark against which more sophisticated methods can be compared. Moreover, as we show below, the results provided by these methods are good enough to enable biologists to conduct targeted laboratory experiments.

Each of the five methods is discriminative. That is, the classifiers learned by the methods assign a real number (called a discriminant value) to each gene, reflecting the classifier's certainty that the gene responds to stress. Formally, a discriminative classifier is a function, f^
 MathType@MTEF@5@5@+=feaafiart1ev1aaatCvAUfKttLearuWrP9MDH5MBPbIqV92AaeXatLxBI9gBaebbnrfifHhDYfgasaacH8akY=wiFfYdH8Gipec8Eeeu0xXdbba9frFj0=OqFfea0dXdd9vqai=hGuQ8kuc9pgc9s8qqaq=dirpe0xb9q8qiLsFr0=vr0=vr0dc8meaabaqaciaacaGaaeqabaqabeGadaaakeaacuWGMbGzgaqcaaaa@2E11@, from genes to discriminant values. In our case, each gene is represented as a 290-dimensional vector, **x**, whose components are the expression levels of the gene under the 290 experimental conditions. Thus, if **x **is a vector representing a gene, then *dv *= f^
 MathType@MTEF@5@5@+=feaafiart1ev1aaatCvAUfKttLearuWrP9MDH5MBPbIqV92AaeXatLxBI9gBaebbnrfifHhDYfgasaacH8akY=wiFfYdH8Gipec8Eeeu0xXdbba9frFj0=OqFfea0dXdd9vqai=hGuQ8kuc9pgc9s8qqaq=dirpe0xb9q8qiLsFr0=vr0=vr0dc8meaabaqaciaacaGaaeqabaqabeGadaaakeaacuWGMbGzgaqcaaaa@2E11@(**x**) is the discriminant value assigned to the gene by the classifier. Finally, a decision threshold, *τ*, is chosen, and the gene is predicted to respond to stress if and only if *dv *> *τ*.

#### Unsupervised, semi-supervised and transductive learning

In addition to these supervised learning methods, we preprocessed the gene expression data using Principal Components Analysis (PCA), a form of unsupervised learning, to reduce the dimensionality of the data (see Methods). For this purpose, we combined the expression-level measurements for all genes (both annotated and unannotated) into one large data set, and applied PCA to the entire set. We are therefore doing a form of semi-supervised learning [29,30], in which unsupervised learning uses the entire data set (ignoring annotations), and then supervised learning uses the annotated data. This increases the effectiveness of learning by increasing the amount of training data used in the unsupervised phase [29,30]. In our case, the unannotated data is also the prediction data, which means that information about the prediction data is used during (unsupervised) training. This is possible because we know all the prediction data in advance. That is, we know the expression levels for all the genes in *Arabidopsis *whether they are annotated or not. We are therefore doing a form of transductive learning [29,31], in which the entire prediction set is known during training and is exploited to predict its annotations. This has the added computational advantage of simplifying the way PCA is done during cross validation (see Methods).

### Estimating classifier performance

To evaluate the performance of discriminative classifiers, it is common to use receiver operating characteristic (ROC) curves [32]. A ROC curve plots the true positive rate (TP) of a classifier against the false positive rate (FP) for various decision thresholds. It therefore shows the quality of a classifier not at one threshold, but at many, and provides more information than a simple miss-classification rate (as in [33] for example). In practice, however, biologists are not usually interested in having more than a few dozen false positives, especially in unbalanced data such as ours, in which the number of false positives can rapidly overwhelm the number of true positives. We therefore use so-called ROC_50 _curves [34], a variant of ROC curves in which the horizonal axis only goes up to 50 false positives. The area under a ROC_50 _curve is the ROC_50 _score [34], and is a measure of the overall usefulness of a classifier.

**Figure 1:**
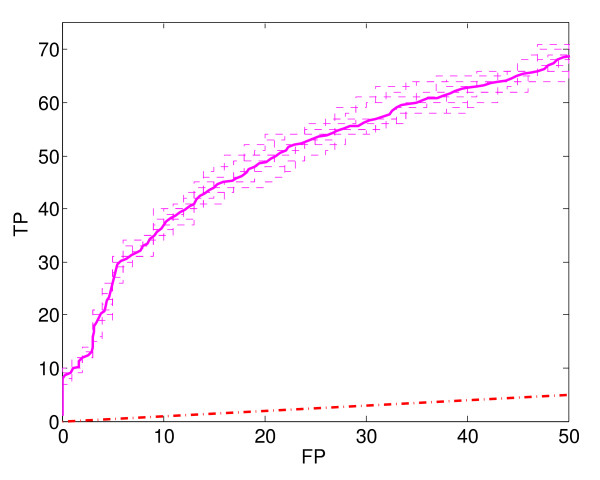
**ROC_50 _curves**. Estimated ROC_50 _curves of the combined classifier (WV), showing ten different estimates (dashed curves) and their average (solid curve).

**Figure 2:**
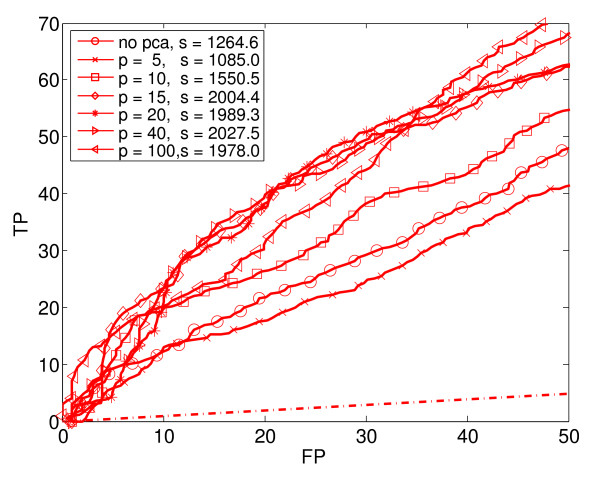
**Logistic Regression (LR)**. Seven ROC_50 _curves for Logistic Regression with varying amounts of dimensionality reduction using PCA. In the legend, p is the PCA-reduced dimension, and s is the ROC_50 _score.

**Figure 3:**
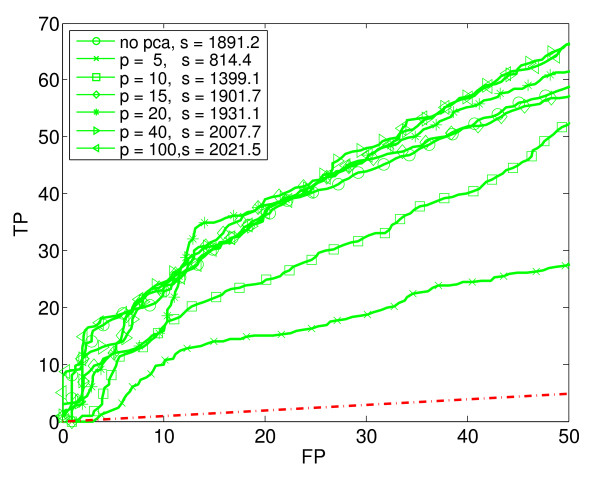
**Linear Discriminant Analysis (LDA)**. Seven ROC_50 _curves for Linear Discriminant Analysis with varying amounts of dimensionality reduction using PCA. In the legend, p is the PCA-reduced dimension, and s is the ROC_50 _score.

**Figure 4:**
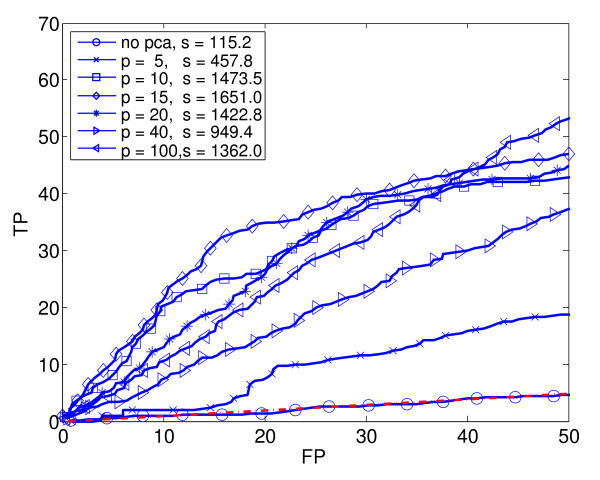
**Quadratic Discriminant Analysis (QDA)**. Seven ROC_50 _curves for Quadratic Discriminant Analysis with varying amounts of dimensionality reduction using PCA. In the legend, p is the PCA-reduced dimension, and s is the ROC_50 _score.

**Figure 5:**
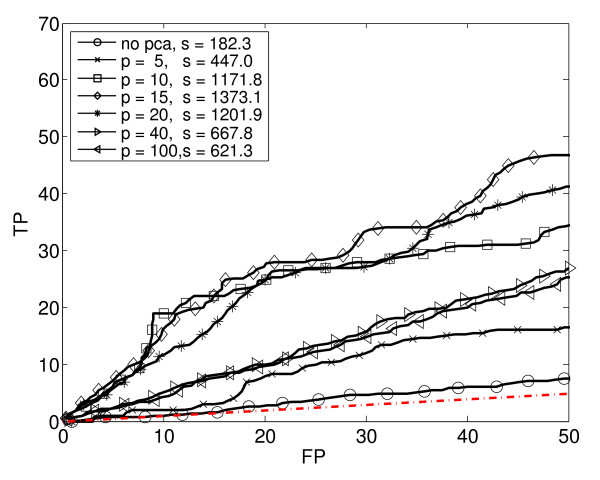
**Naive Bayes (NB)**. Seven ROC_50 _curves for Naive Bayes with varying amounts of dimensionality reduction using PCA. In the legend, p is the PCA-reduced dimension, and s is the ROC_50 _score.

**Figure 6:**
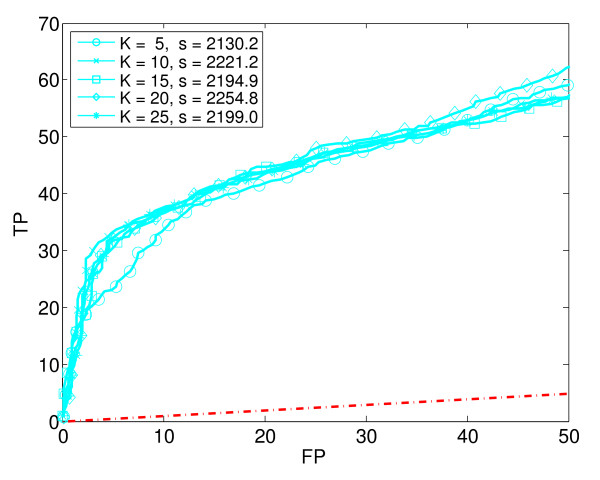
**K-Nearest Neighbours (KNN)**. Five ROC_50 _curves for K-Nearest Neighbours for various values of K. The legend gives the ROC_50 _score, s, for each value of K.

**Figure 7:**
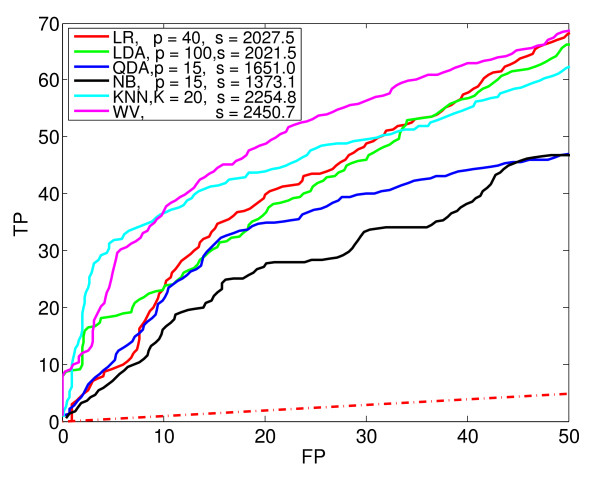
**Comparison of methods**. The ROC_50 _curve (purple) for the combined classifier using weighted voting (WV), and the best ROC_50 _curves from each of Figures 2 to 6. In the legend, p is the PCA-reduced dimension of the data, and s is the ROC_50 _score.

To estimate ROC_50 _curves for our classifiers, we used 20-fold cross-validation (see Methods). Because cross-validation relies on a random split of the training data into folds (20 folds in our case), there is a certain randomness to the estimated ROC_50 _curve. To provide more accurate results, we performed cross-validation ten times, each time with a different (randomly selected) 20-fold split of the data (see Methods). Each 20-fold split results in a slightly different ROC_50 _curve. In some cases, we plot all ten of these curves, to give an idea of the uncertainty in classifier performance (Figure 1). In cases where this would result in overly cluttered graphs, we simply present the average of the ten ROC_50 _curves (Figures 2 to 7, each of which show several average ROC_50 _curves).

We generated ROC_50 _curves for each supervised learning method combined with various amounts of dimensionality reduction. Using PCA, we reduced the original 290 dimensions to 5, 10, 15, 20, 40 and 100 dimensions, respectively. In this way, the original data set was transformed into six separate data sets of various dimensions. Each basic learning method (except KNN) was applied to the original data set and to each of the six reduced data sets. Thus, for each basic learning method (except KNN), we trained and tested seven different classifiers. In the case of KNN, we used only the original, unreduced data, but with five different values of K. Altogether, we trained and tested a total of 4 × 7 + 5 = 33 different classifiers. Figures 2 to 6 show the estimated performance of these basic classifiers. Each figure shows a number of ROC_50 _curves, each derived using cross-validation averaged over a number of random splits of the data, as described above. Unlike traditional ROC curves, the axes of these curves give the number of true and false positives, instead of the proportion. The red dash-dot line near the bottom of each figure shows the expected performance of a random classifier (*i.e*., a classifier that ignores the expression data and guesses whether or not a gene responds to stress by essentially flipping a coin). The ROC_50 _scores for the curves are shown in the legend of each figure.

As the figures show, in some cases the classifiers perform not much better than random, but in most cases they perform significantly better. The figures also show that the performance of each classification method depends heavily of the amount of dimensionality reduction used. Notice in particular that in some cases, the classifier trained on the reduced data has a much higher ROC_50 _score than the classifier trained on the original, unreduced data. This is especially true for NB and QDA. For instance, the classifiers trained on the original data have low ROC_50 _scores of 182.3 for NB and 115.2 for QDA. This is comparable to the random classifier, whose ROC_50 _score is 122.5. However, reducing the dimensionality of the data to 15 increases their ROC_50 _scores to 1373.1 and 1651.0, respectively. This shows the importance of dimensionality reduction. In contrast, KNN performs well for all the values of K that we used.

Figure 7 compares the basic classification methods by plotting the best performance of each. That is, for each of the basic classification methods, the ROC_50 _curve with the highest ROC_50 _score is reproduced in Figure 7. In addition, the figure shows the performance of a classification method that uses a weighted voting scheme (WV) to combine the 33 basic classifiers into a single, composite classifier. Notice that this composite classifier performs best of all. The next section describes how this composite classifier is constructed.

### Improving prediction accuracy by combining classifiers

Combining different classifiers in prediction can be thought of as combining different opinions in decision making. The advantage is that a group opinion is better than a single opinion if the single opinions are correctly weighted and combined. In machine-learning systems, classifiers are often combined by weighted voting, in which the discriminant value of the combined classifier is a linear combination of the discriminant values of the individual classifiers. Formally, given a set of basic classifiers, f^1,…,f^M
 MathType@MTEF@5@5@+=feaafiart1ev1aaatCvAUfKttLearuWrP9MDH5MBPbIqV92AaeXatLxBI9gBaebbnrfifHhDYfgasaacH8akY=wiFfYdH8Gipec8Eeeu0xXdbba9frFj0=OqFfea0dXdd9vqai=hGuQ8kuc9pgc9s8qqaq=dirpe0xb9q8qiLsFr0=vr0=vr0dc8meaabaqaciaacaGaaeqabaqabeGadaaakeaacuWGMbGzgaqcamaaBaaaleaacqaIXaqmaeqaaOGaeiilaWIaeS47IWKaeiilaWIafmOzayMbaKaadaWgaaWcbaGaemyta0eabeaaaaa@3599@, and a set of weights, *w*_1_, …, *w*_*M*_, the combined classifier, f^
 MathType@MTEF@5@5@+=feaafiart1ev1aaatCvAUfKttLearuWrP9MDH5MBPbIqV92AaeXatLxBI9gBaebbnrfifHhDYfgasaacH8akY=wiFfYdH8Gipec8Eeeu0xXdbba9frFj0=OqFfea0dXdd9vqai=hGuQ8kuc9pgc9s8qqaq=dirpe0xb9q8qiLsFr0=vr0=vr0dc8meaabaqaciaacaGaaeqabaqabeGadaaakeaacuWGMbGzgaqcaaaa@2E11@, is defined by the equation f^(x)=∑mwmf^m(x)
 MathType@MTEF@5@5@+=feaafiart1ev1aaatCvAUfKttLearuWrP9MDH5MBPbIqV92AaeXatLxBI9gBaebbnrfifHhDYfgasaacH8akY=wiFfYdH8Gipec8Eeeu0xXdbba9frFj0=OqFfea0dXdd9vqai=hGuQ8kuc9pgc9s8qqaq=dirpe0xb9q8qiLsFr0=vr0=vr0dc8meaabaqaciaacaGaaeqabaqabeGadaaakeaacuWGMbGzgaqcaiabcIcaOGqabiab=Hha4jabcMcaPiabg2da9maaqababaGaem4DaC3aaSbaaSqaaiabd2gaTbqabaGccuWGMbGzgaqcamaaBaaaleaacqWGTbqBaeqaaOGaeiikaGIae8hEaGNaeiykaKcaleaacqWGTbqBaeqaniabggHiLdaaaa@3EC3@. In our case, *M *= 33, as described above.

By judiciously choosing the weights, *w*_1_, …, *w*_*M*_, the performance of the combined classifier can be maximized. Various methods are available for doing this, such as model averaging and stacking [21]. Using these methods on our data sets, we found that the ROC curve of the combined classifier was usually better than the ROC curves of the basic classifiers, as expected. Unfortunately, we also found that the ROC_50 _curve of the combined classifier was usually worse (data not shown). We hypothesized that this is because our data sets are highly unbalanced. Intuitively, model averaging and stacking try to choose weights so as to correctly classify as much data as possible. In our case, this means trying to correctly classify the vast number of negative samples in our data sets, even if this means misclassifying the small number of positives. In other words, these methods try to minimize the total number of false positives, even though we only care about the first fifty.

To choose appropriate weights for our combined classifier, we used the heuristic that classifiers that perform well should be given more weight than classifiers that perform poorly. In our case, since we want to maximize the ROC_50 _score of the combined classifier, we want to give high weight to classifiers with high ROC_50 _scores. There are many ways to do this, but we found that it was sufficient to estimate and normalize the ROC_50 _score of each basic classifier, and use this as its weight. That is, we used wm=s^m/∑ms^m
 MathType@MTEF@5@5@+=feaafiart1ev1aaatCvAUfKttLearuWrP9MDH5MBPbIqV92AaeXatLxBI9gBaebbnrfifHhDYfgasaacH8akY=wiFfYdH8Gipec8Eeeu0xXdbba9frFj0=OqFfea0dXdd9vqai=hGuQ8kuc9pgc9s8qqaq=dirpe0xb9q8qiLsFr0=vr0=vr0dc8meaabaqaciaacaGaaeqabaqabeGadaaakeaacqWG3bWDdaWgaaWcbaGaemyBa0gabeaakiabg2da9iqbdohaZzaajaWaaSbaaSqaaiabd2gaTbqabaGccqGGVaWldaaeqaqaaiqbdohaZzaajaWaaSbaaSqaaiabd2gaTbqabaaabaGaemyBa0gabeqdcqGHris5aaaa@3B09@, where s^m
 MathType@MTEF@5@5@+=feaafiart1ev1aaatCvAUfKttLearuWrP9MDH5MBPbIqV92AaeXatLxBI9gBaebbnrfifHhDYfgasaacH8akY=wiFfYdH8Gipec8Eeeu0xXdbba9frFj0=OqFfea0dXdd9vqai=hGuQ8kuc9pgc9s8qqaq=dirpe0xb9q8qiLsFr0=vr0=vr0dc8meaabaqaciaacaGaaeqabaqabeGadaaakeaacuWGZbWCgaqcamaaBaaaleaacqWGTbqBaeqaaaaa@2FBA@ is an estimate of the ROC_50 _score of classifier *f*_*m*_. Note that with these weights, if each f^m
 MathType@MTEF@5@5@+=feaafiart1ev1aaatCvAUfKttLearuWrP9MDH5MBPbIqV92AaeXatLxBI9gBaebbnrfifHhDYfgasaacH8akY=wiFfYdH8Gipec8Eeeu0xXdbba9frFj0=OqFfea0dXdd9vqai=hGuQ8kuc9pgc9s8qqaq=dirpe0xb9q8qiLsFr0=vr0=vr0dc8meaabaqaciaacaGaaeqabaqabeGadaaakeaacuWGMbGzgaqcamaaBaaaleaacqWGTbqBaeqaaaaa@2FA0@(**x**) is a number between 0 and 1 (as with our classifiers), then so is f^
 MathType@MTEF@5@5@+=feaafiart1ev1aaatCvAUfKttLearuWrP9MDH5MBPbIqV92AaeXatLxBI9gBaebbnrfifHhDYfgasaacH8akY=wiFfYdH8Gipec8Eeeu0xXdbba9frFj0=OqFfea0dXdd9vqai=hGuQ8kuc9pgc9s8qqaq=dirpe0xb9q8qiLsFr0=vr0=vr0dc8meaabaqaciaacaGaaeqabaqabeGadaaakeaacuWGMbGzgaqcaaaa@2E11@(**x**). Also, this method automatically gives low weight to classifiers that use an inappropriate amount of dimensionality reduction, since such classifiers have low ROC_50 _scores. In this way, the combined classifier incorporates not only the best combination of supervised learning methods, but also the best amounts of dimensionality reduction for each method.

To train and evaluate the combined classifier, we used *two *sets of validation data. After the basic classifiers were trained, one validation set was used to estimate their ROC_50 _scores. The combined classifier was then constructed using these scores, and the second validation set was used to estimate its ROC_50 _curve. Thus, the validation data for the basic classifiers is part of the training data for the combined classifier. To do this in a cross-validation setting, we used what amounts to nested cross-validation (see Methods). As shown in Figure 7, the resulting combined classifier has a higher ROC_50 _score than any of the basic classifiers from which it is made.

Figure 1 gives another view of the performance of the combined classifier. Here, the thin dashed lines are a superposition of ten different curves, where each one is a different estimate of the combined classifier's true ROC_50 _curve. As described earlier, each estimate of a classifier's ROC_50 _curve includes some randomness, due to the random choice of folds during cross-validation. The ten dashed curves in Figure 1 are derived from ten different cross-validations, each one using a different set of folds. The thick solid line in the figure is the average of the other ten curves. Because averaging reduces variance, the average curve is a more accurate estimate of the true ROC_50 _curve (i.e., has lower variance) than any of the other ten curves. The diagonal dash-dot line near the bottom of the plot shows the expected performance of a random classifier.

ROC and ROC_50 _curves plot the number of true positives against the number of false positives. However, in applications such as ours, the *precision *is also of interest. Precision is the proportion of true positives (TP) among the predicted positives (PP). (It is also the complementary false discovery rate, 1-FDR [35].) Precision is important since each prediction is a potential experiment, and as a matter of economics, a biologist needs an estimate of how many of the experiments will succeed. This is especially important in situations, such as ours, where the number of real negatives is much greater than the number of real positives, and so there is a real possibility of having a huge number of failed experiments.

**Figure 8:**
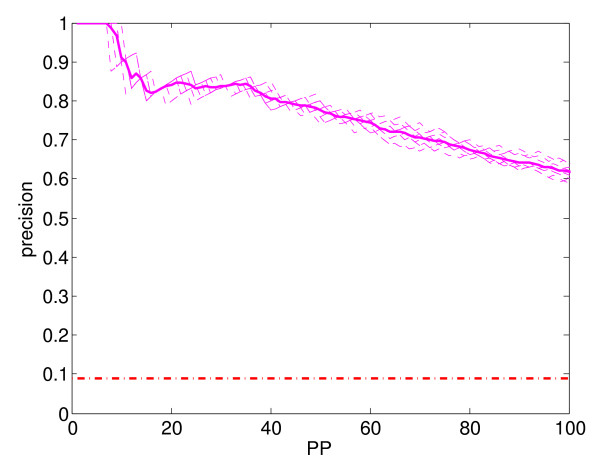
**Precision curves**. Estimated precision curves of the combined classifier (WV), showing ten different estimates (dashed curves) and their average (solid curve).

Figure 8 plots estimated precision against the number of predictions for the first hundred predictions. Notice that as the number of predictions increases (*i.e*., as the classifier's decision threshold is lowered), the precision decreases, meaning that fewer of the predictions are expected to be true. As in Figure 1, the thin dashed lines are a superposition of ten different curves, each one an estimate of the true precision curve, and the thick solid line is their average. Also, the horizontal dash-dot line near the bottom of the plot is the expected precision of a random classifier, and its height is equal to the ratio of the number of positives (*i.e*., stress genes) to the total number of samples (*i.e*., genes) in the training data. Since all the estimated precision curves are well above the horizontal dash-dot line, the performance of the combined classifier for the first hundred predictions is significantly better than random. Also, since Figures 1 and 8 show small variance, and since the variance of the average curves will be even less, the combined classifier should have stable prediction performance.

### Stress-response predictions

We trained the combined classifier on our Arabidopsis data set, using all 22,746 genes for Principal Components Analysis, and the 11,553 annotated genes for supervised learning, as described above. We then applied the classifier to the 11,193 unannotated genes, to get a set of 11,193 predictions (see Methods). Table 1 shows the top fifty predictions. Each row in the table is a prediction: the first (leftmost) entry is the rank of the prediction (1 being the top prediction); the second entry identifies a gene; the third entry is a discriminant value (measuring the likelihood that the gene responds to stress); and the fourth entry is the estimated precision of the prediction and all predictions above it (*i.e*., the fraction of these predictions expected to be true). As an example, consider the 23rd row of the table, the row for gene At1g09950. Since the estimated precision in this row is given as 0.7044, we expect that about 70% of the top 23 genes respond to stress, *i.e*., 16 genes.

**Table 1:** The top 50 predictions of the combined classifier ordered by discriminant value

No.	Gene name	Dv	Pr
1	At1g61340	0.7879	0.8491
2	At1g72660	0.7315	0.8423
3	At5g04340	0.7269	0.8405
4	At1g19180	0.7219	0.8448
5	At2g01520	0.7017	0.8311
6	At2g36220	0.6987	0.8293
7	At5g10695	0.6912	0.8138
8	At3g10020	0.6850	0.8030
9	At3g16050	0.6778	0.8000
10	At4g18280	0.6673	0.7945
11	At1g11210	0.6636	0.7955
12	At5g64510	0.6514	0.7900
13	At3g09350	0.6412	0.7807
14	At5g42380	0.6357	0.7718
15	At3g44860	0.6278	0.7623
16	At1g73260	0.6252	0.7583
17	At1g16850	0.6186	0.7452
18	At1g78070	0.6185	0.7439
19	At3g01830	0.6098	0.7398
20	At5g19875	0.6094	0.7402
21	At3g62260	0.6040	0.7213
22	At1g03070	0.5961	0.7106
23	At1g09950	0.5942	0.7044
24	At1g19020	0.5867	0.6928
25	At1g07430	0.5866	0.6919
26	At1g76960	0.5860	0.6901
27	At1g30070	0.5838	0.6819
28	At2g05510	0.5799	0.6726
29	At3g50930	0.5796	0.6726
30	At1g67360	0.5767	0.6691
31	At5g09530	0.5758	0.6703
32	At3g53230	0.5737	0.6663
33	At3g55970	0.5694	0.6586
34	At4g27657	0.5676	0.6549
35	At4g38080	0.5658	0.6458
36	At1g17380	0.5651	0.6448
37	At4g27652	0.5647	0.6445
38	At1g68500	0.5588	0.6204
39	At1g76650	0.5573	0.6146
40	At2g15960	0.5549	0.6074
41	At1g14870	0.5520	0.6017
42	At1g49450	0.5497	0.5991
43	At1g13930	0.5467	0.5942
44	At2g32190	0.5453	0.5914
45	At4g23493	0.5429	0.5879
46	At2g28400	0.5418	0.5842
47	At1g48720	0.5399	0.5780
48	At3g02480	0.5384	0.5721
49	At2g43620	0.5376	0.5677
50	At4g14270	0.5373	0.5676

Pr, estimated precision; Dv, discriminant value.

**Figure 9:**
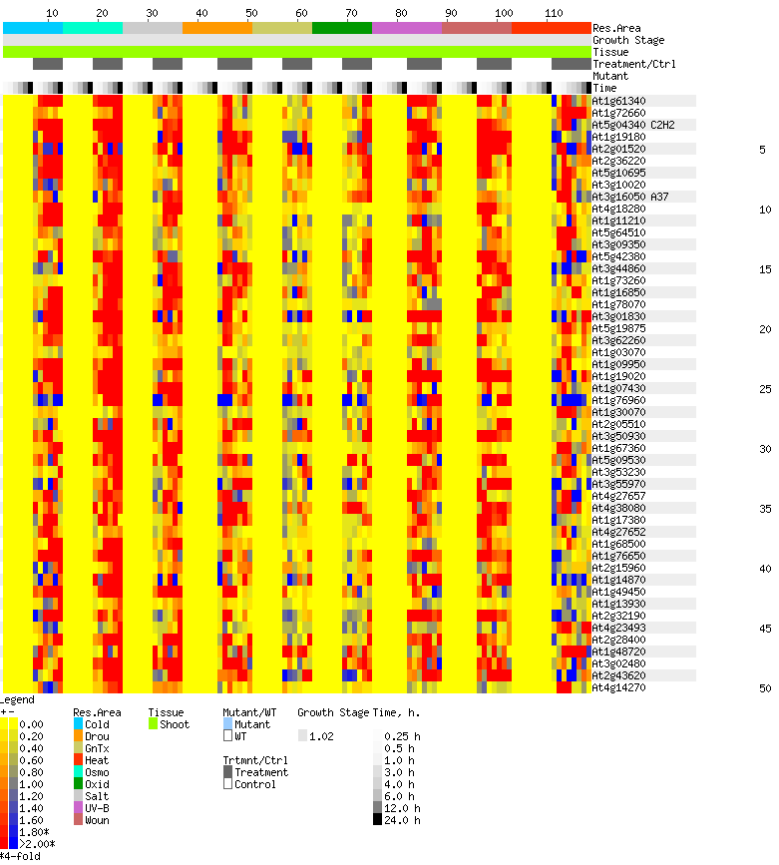
**Electronic Northern analysis**. E-Northern of the top 50 predictions.

**Figure 10:**
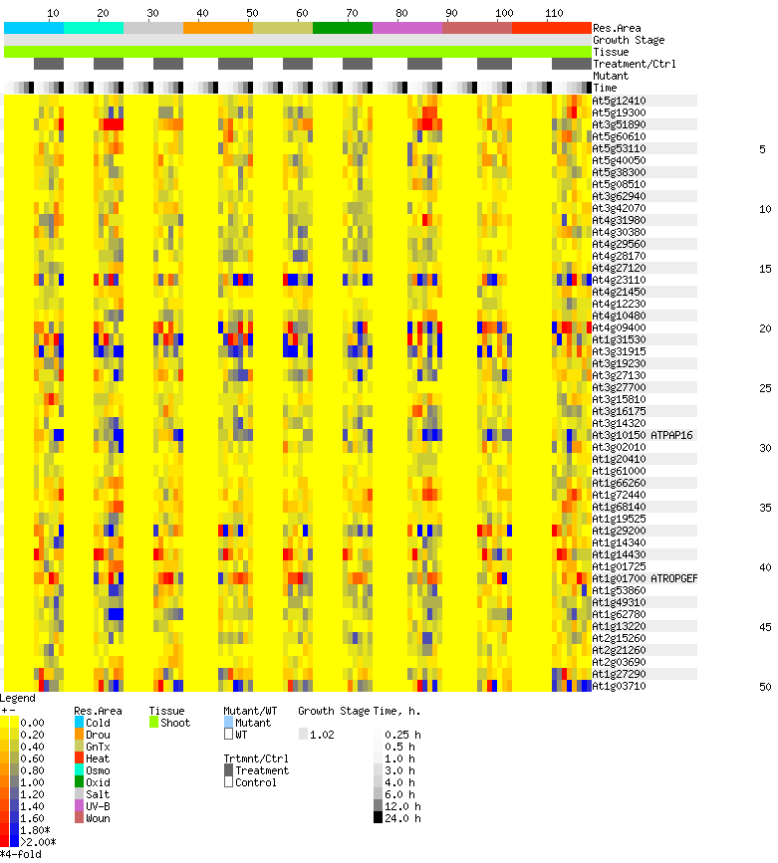
**Electronic Northern analysis**. E-Northern of 50 randomly selected genes.

Figures 9 and 10 provide visual evidence supporting these predictions. Each figure shows a heat map. These maps, known as "electronic Northerns" (or e-Northerns), were generated using the Expression Browser tool of the Botany Array Resource (BAR) and the AtGenExpress Stress Series (shoot) data set[23]. The program contains expression data for more than 22,000 genes across more than 1000 samples collected from NASCArrays, AtGenExpress Consortium, and the Department of Botany at the University of Toronto [22-24,36]. Each row in an e-Northern is a gene, and each column is an experiment. The colour at a point represents the relative expression level of the gene during the experiment. More specifically, the colour represents the log_2 _of the ratio of the average of replicate treatments relative to the average of corresponding controls. Yellow means that under the experimental conditions, the gene had the same expression level as the control. (The wide, yellow vertical stripes are the controls.) Red means that the gene had a higher expression level than the control (up-regulation), and blue means it had a lower expression level (down-regulation). A gene that shows significant up-regulation (or down-regulation) under stress conditions is likely to be involved in response to stress. Thus, unlike cross validation, electronic Northerns provide a means of evaluating the quality of predictions based on the prediction data, not just the training data. The e-Northerns of Figures 9 and 10, for instance, are based entirely on prediction data. In these e-Northerns, the experiments exposed the plant to various stress conditions, such as heat, cold, drought, UV-B radiation, etc. Figure 9 is the e-Northern for the top-50 predictions of our combined classifier, *i.e*., for the 50 genes predicted to most likely to respond to stress. For comparison, Figure 10 is the e-Northern for 50 genes chosen at random from the prediction set. Note that there is much more colour in Figure 9 than in Figure 10, especially red. This suggests that our combined classifier has indeed extracted meaningful gene expression patterns for genes that respond to stress.

### Gene knockout experiments

From the predictions of the combined classifier, three genes were chosen for biological analysis using gene knockout experiments. Here, we present the results for one of these genes, At1g16850, which show it to be necessary for the normal response to temperature and NaCl. Our results also confirm that the other two genes, At1g11210 and At4g39675, are involved in a variety of stress responses (data not shown).

The criteria used to choose candidate genes for subsequent biological analysis were: 1) the gene must be expressed in either root or shoot, 2) gene expression should be strongly increased in response to abiotic stress, such as cold, drought, osmotic and salt stresses, 3) T-DNA knockout lines – in which a given gene's expression has been eliminated – should available from the Salk Institute [37], and 4) the gene should not have an annotated function nor be present in any patent database. Further bioinformatics analysis was performed using Athena for promoter motif prediction [38], Expression Angler for co-expressed gene analysis [22] and eFP browser for electronic representation of gene expression patterns [39].

#### Stress response

**Figure 11:**
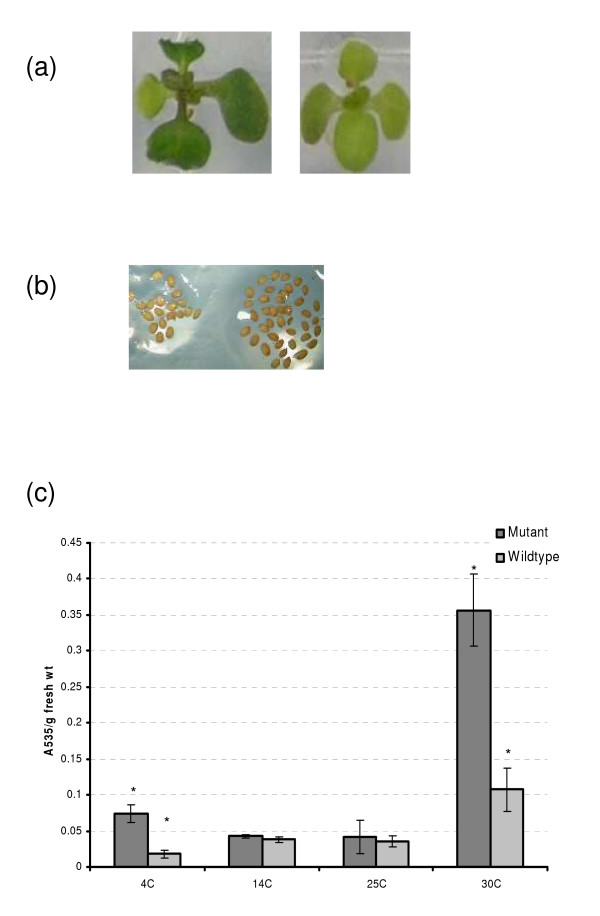
**Gene knockout experiments**. 10 day old wild-type and mutant plants after exposure for 7 days at 14. (a) The mutant cotyledons appear darker than wild-type due to increased anthocyanin levels. (b) mutant and wild-type seeds 24 h after sowing on agar plates. Mutant seeds have the appearance of lighter colour compared to wild-type. (c) Quantification of anthocyanin levels measuring A535. Bars indicate standard error of 5 replicate measurements. * indicates significantly different at *p *< 0.05

The increased presence of anthocyanin levels in plants lacking a functional copy of the At1g16850 gene during cold stress of 4C indicates that this gene is involved in cold stress response (Figure 11). The same effect is seen at 30C, indicating that this gene is also associated with response to heat stress (Figure 11). Interestingly, At1g16850 is normally expressed during the later stages of seed maturation, towards seed dessication, and hence may play a role in seed dormancy. This sort of bifunctionality is seen with other stress response genes, which have documented roles in the cold, heat and salt stress pathways, e.g. RD29A (Response to Desiccation) and LEA (Late Embryogensis Abundant) protein [40,41]. These proteins have also been found to accumulate during seed maturation [40,41] and are in fact co-expressed with At1g16850 under stress conditions and during seed maturation, as determined using the Expression Angler algorithm [22].

**Figure 12:**
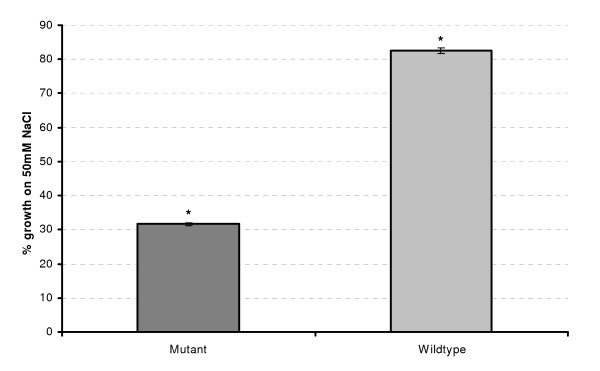
**Gene knockout experiments**. Root growth on 50 mM NaCl, relative to growth on 0 mM NaCl, on 10 day old wild-type and mutant plants transferred to 50 mM NaCl medium. Error bars indicate the standard error of 5 replicates. *n *= 25 measurements per treatment and genotype. * indicates significantly different at *p *< 0.001

In addition to modulating a response to temperature, plants lacking a functional At1g16850 exhibit a defective root growth phenotype under increasing salt concentrations (Figure 12). This phenotype, combined with previous microarray studies [42], which found At1g16850 induction at 250 mM NaCl, gives clear indication that At1g16850 is also part of the salt stress response pathway.

## Conclusion

In this study, we evaluated and compared five basic supervised learning methods (LR, LDA, QDA, NB and KNN) for gene function prediction in *A. thaliana *based solely on gene expression data. The major advantage of supervised methods over unsupervised methods is that by including prior knowledge of class information, supervised methods can ignore uninformative features and select informative features that are useful for separating classes. In this study, we focussed on finding genes that respond to stress, as represented by the term *GO:0006950 [response to stress] *in the GOBP hierarchy. Using a training set of genes of known function, we used the basic learning methods to predict the stress response of genes of unknown function. We estimated the accuracy of the predictions using ROC_50 _scores derived through cross validation. We found, for instance, that KNN performs well for various values of K. For the other learning methods, the performance depends greatly on whether the data is preprocessed using PCA, and on how much its dimensionality is reduced. Using various values of K and various amounts of dimensionality reduction, we trained and tested a total of 33 basic classifiers.

We also investigated combining the basic classifiers using weighted voting. Our method of constructing the combined classifier chooses not only the best combination of supervised learning methods, but also the best amount of dimensionality reduction for each method. Our results show that the combined classifier outperforms all the basic classifiers in predicting whether a gene responds to stress. This can be attributed to the relative robustness of methods for combining classifiers. Intuitively, any single learning method represents a single view of the data, while a combination method represents multiple views strategically combined. The proper choice of combining method is important to the success of a combined classifier. For example, model averaging and stacking are well-known methods for combining classifiers [21]; however, we found that while they did improve on the overall ROC curves of the basic classifiers, the ROC_50 _curve was often worse (data not shown). In contrast, our weighted voting method using ROC_50 _scores as weights is simple, provides improved accuracy in predicting stress response in *A. thaliana*, and we would expect it to provide improved accuracy in other organisms and for other gene functions.

Using electronic Northern analysis, we observed significant up-regulation and down-regulation of many of our predictions. The strong up- and down-regulation are also present among the stress-response genes in the training data (data not shown). In contrast, randomly selected genes show much less up- and down-regulation. This visually confirms that the combined classifier is able to distinguish between stress and non-stress genes. Moreover, unlike cross-validation, this confirmation is based on the prediction data, not the training data.

Using gene knockout experiments – in which a given gene's expression is eliminated – we tested three of our predictions. We presented the results for one of these genes, At1g16850, which show it to be involved in the stress response pathways to cold (4C), chill (14C) and NaCl. We have also confirmed the biological stress responsive roles of the other two genes, At1g11210 and At4g39675 (data not shown). Further biological studies will determine the pattern of expression in specific cell and tissues types of the plant and the exact physiological role of these genes.

## Methods

### Preprocessing of raw gene expression data

The gene expression data from the Botany Array Resource at the University of Toronto contain *detection calls*: P (present), M (marginal) and A (absent). The detection call determines whether a transcript is reliably detected (present), partially detected (marginal), or not detected (absent). The following is an example for the gene *At3g24440 *under three selected conditions:

AT3G24440 : 243.10 P : 120.90 A : 109.40 M

We simply removed these detection calls (P, A, and M) in this study. In addition, gene expression levels were log transformed. The transformed data have approximately normal distributions while the raw data have approximately exponential distributions (data not shown). Many of the learning methods used in this study were designed with normal data in mind.

### Basic supervised learning methods

Each of the learning methods described below trains a discriminative classifier. We used the methods to train binary classifiers in which the two classes correspond to genes that respond to stress (Class 1) and genes that do not (Class 0). Given a vector, **x**, of gene expression measurements, each classifier returns a discriminant value, *dv*(**x**), reflecting the classifier's confidence that the gene belongs to Class 1. The gene is assigned to Class 1 if and only if *dv*(**x**) > *τ*, where *τ *is a decision threshold. For the classifiers LR, LDA, QDA and NB, the discriminate value is an estimate of *p*(*k *= 1|**x**), the posterior probability that the gene is in Class 1. For KNN, the discriminant value is simply a number between 0 and 1.

#### LR (Logistic Regression)

Given a set of classes, LR models the log likelihood ratio for any pair of classes as a linear function of the test vector, **x**, and thus defines linear decision boundaries between the classes. In the case of just two classes, the model has the simple form

logp(k=1|x)p(k=0|x)=β0+β1Tx
 MathType@MTEF@5@5@+=feaafiart1ev1aaatCvAUfKttLearuWrP9MDH5MBPbIqV92AaeXatLxBI9gBaebbnrfifHhDYfgasaacH8akY=wiFfYdH8Gipec8Eeeu0xXdbba9frFj0=OqFfea0dXdd9vqai=hGuQ8kuc9pgc9s8qqaq=dirpe0xb9q8qiLsFr0=vr0=vr0dc8meaabaqaciaacaGaaeqabaqabeGadaaakeaaieGacqWFSbaBcqWFVbWBcqWFNbWzdaWcaaqaaiabdchaWjabcIcaOiabdUgaRjabg2da9iabigdaXiabcYha8Hqabiab+Hha4jabcMcaPaqaaiabdchaWjabcIcaOiabdUgaRjabg2da9iabicdaWiabcYha8jab+Hha4jabcMcaPaaacqGH9aqpiiGacqqFYoGydaWgaaWcbaGaeGimaadabeaakiabgUcaRiab9j7aInaaDaaaleaacqaIXaqmaeaacqWGubavaaGccqGF4baEaaa@4DC2@

and hence,

p(k=1|x)=eβ0+β1Tx1+eβ0+β1Tx
 MathType@MTEF@5@5@+=feaafiart1ev1aaatCvAUfKttLearuWrP9MDH5MBPbIqV92AaeXatLxBI9gBaebbnrfifHhDYfgasaacH8akY=wiFfYdH8Gipec8Eeeu0xXdbba9frFj0=OqFfea0dXdd9vqai=hGuQ8kuc9pgc9s8qqaq=dirpe0xb9q8qiLsFr0=vr0=vr0dc8meaabaqaciaacaGaaeqabaqabeGadaaakeaacqWGWbaCcqGGOaakcqWGRbWAcqGH9aqpcqaIXaqmcqGG8baFieqacqWF4baEcqGGPaqkcqGH9aqpdaWcaaqaaiabdwgaLnaaCaaaleqabaacciGae4NSdi2aaSbaaWqaaiabicdaWaqabaWccqGHRaWkcqGFYoGydaqhaaadbaGaeGymaedabaGaemivaqfaaSGae8hEaGhaaaGcbaGaeGymaeJaey4kaSIaemyzau2aaWbaaSqabeaacqGFYoGydaWgaaadbaGaeGimaadabeaaliabgUcaRiab+j7aInaaDaaameaacqaIXaqmaeaacqWGubavaaWccqWF4baEaaaaaaaa@4E33@

p(k=0|x)=11+eβ0+β1Tx
 MathType@MTEF@5@5@+=feaafiart1ev1aaatCvAUfKttLearuWrP9MDH5MBPbIqV92AaeXatLxBI9gBaebbnrfifHhDYfgasaacH8akY=wiFfYdH8Gipec8Eeeu0xXdbba9frFj0=OqFfea0dXdd9vqai=hGuQ8kuc9pgc9s8qqaq=dirpe0xb9q8qiLsFr0=vr0=vr0dc8meaabaqaciaacaGaaeqabaqabeGadaaakeaacqWGWbaCcqGGOaakcqWGRbWAcqGH9aqpcqaIWaamcqGG8baFieqacqWF4baEcqGGPaqkcqGH9aqpdaWcaaqaaiabigdaXaqaaiabigdaXiabgUcaRiabdwgaLnaaCaaaleqabaacciGae4NSdi2aaSbaaWqaaiabicdaWaqabaWccqGHRaWkcqGFYoGydaWgaaadbaacbaGae0xmaedabeaaliab=Hha4baaaaaaaa@4356@

and *p*(*k *= 1|**x**) + *p*(*k *= 0|**x**) = 1. The parameters *β*_0 _and *β*_1 _are fitted to the training data using maximum likelihood [21].

#### LDA (Linear Discriminant Analysis)

LDA models the classes as multivariate Gaussians, where each class is assumed to have the same covariance matrix. The density function for class *k *is therefore given by

gk(x)=1(2π)p/2|Σ|1/2e−(x−μk)TΣ−1(x−μk)/2
 MathType@MTEF@5@5@+=feaafiart1ev1aaatCvAUfKttLearuWrP9MDH5MBPbIqV92AaeXatLxBI9gBaebbnrfifHhDYfgasaacH8akY=wiFfYdH8Gipec8Eeeu0xXdbba9frFj0=OqFfea0dXdd9vqai=hGuQ8kuc9pgc9s8qqaq=dirpe0xb9q8qiLsFr0=vr0=vr0dc8meaabaqaciaacaGaaeqabaqabeGadaaakeaacqWGNbWzdaWgaaWcbaGaem4AaSgabeaakiabcIcaOGqabiab=Hha4jabcMcaPiabg2da9maalaaabaGaeGymaedabaGaeiikaGIaeGOmaidcciGae4hWdaNaeiykaKYaaWbaaSqabeaacqWGWbaCcqGGVaWlcqaIYaGmaaGcdaabdaqaaiabfo6atbGaay5bSlaawIa7amaaCaaaleqabaGaeGymaeJaei4la8IaeGOmaidaaaaakiabdwgaLnaaCaaaleqabaGaeyOeI0IaeiikaGIae8hEaGNaeyOeI0Iae4hVd02aaSbaaWqaaiab=TgaRbqabaWccqGGPaqkdaahaaadbeqaaiabdsfaubaaliabfo6atnaaCaaameqabaGaeyOeI0IaeGymaedaaSGaeiikaGIae8hEaGNaeyOeI0Iae4hVd02aaSbaaWqaaiab=TgaRbqabaWccqGGPaqkcqGGVaWlcqaIYaGmaaaaaa@5C4A@

where *μ*_*k *_is the mean vector for class *k*, Σ is the common covariance matrix, and *p *is the dimensionality of **x**. It can be shown [21] that the discriminant function for class *k *is equivalent to the following function:

δk(x)=xTΣ−1μk−12μkTΣ−1μk+logπk
 MathType@MTEF@5@5@+=feaafiart1ev1aaatCvAUfKttLearuWrP9MDH5MBPbIqV92AaeXatLxBI9gBamXvP5wqSXMqHnxAJn0BKvguHDwzZbqegyvzYrwyUfgarqqtubsr4rNCHbGeaGqiA8vkIkVAFgIELiFeLkFeLk=iY=Hhbbf9v8qqaqFr0xc9pk0xbba9q8WqFfeaY=biLkVcLq=JHqVepeea0=as0db9vqpepesP0xe9Fve9Fve9GapdbaqaaeGacaGaaiaabeqaamqadiabaaGcbaacciGae8hTdq2aaSbaaSqaaiabdUgaRbqabaGccqGGOaakimqacaGF4bGaeiykaKIaeyypa0Jaa4hEamaaCaaaleqabaGaemivaqfaaOGaeu4Odm1aaWbaaSqabeaacqGHsislcqaIXaqmaaGccqWF8oqBdaWgaaWcbaGaem4AaSgabeaakiabgkHiTmaalaaabaGaeGymaedabaGaeGOmaidaaiab=X7aTnaaDaaaleaacqWGRbWAaeaacqWGubavaaGccqqHJoWudaahaaWcbeqaaiabgkHiTiabigdaXaaakiab=X7aTnaaBaaaleaacqWGRbWAaeqaaOGaey4kaSccdiGaa0hBaiaa99gacaqFNbGae8hWda3aaSbaaSqaaiabdUgaRbqabaaaaa@624C@

where *π*_*k *_is the prior probability of class *k*. The decision boundaries and therefore linear. The parameters *π*_*k*_, *μ*_*k *_and Σ are estimated by applying maximum likelihood to the training data [21], giving

πk=nkn
 MathType@MTEF@5@5@+=feaafiart1ev1aaatCvAUfKttLearuWrP9MDH5MBPbIqV92AaeXatLxBI9gBaebbnrfifHhDYfgasaacH8akY=wiFfYdH8Gipec8Eeeu0xXdbba9frFj0=OqFfea0dXdd9vqai=hGuQ8kuc9pgc9s8qqaq=dirpe0xb9q8qiLsFr0=vr0=vr0dc8meaabaqaciaacaGaaeqabaqabeGadaaakeaaiiGacqWFapaCdaWgaaWcbaGaem4AaSgabeaakiabg2da9maalaaabaGaemOBa42aaSbaaSqaaiabdUgaRbqabaaakeaacqWGUbGBaaaaaa@357A@

μk=∑xi∈kxink
 MathType@MTEF@5@5@+=feaafiart1ev1aaatCvAUfKttLearuWrP9MDH5MBPbIqV92AaeXatLxBI9gBaebbnrfifHhDYfgasaacH8akY=wiFfYdH8Gipec8Eeeu0xXdbba9frFj0=OqFfea0dXdd9vqai=hGuQ8kuc9pgc9s8qqaq=dirpe0xb9q8qiLsFr0=vr0=vr0dc8meaabaqaciaacaGaaeqabaqabeGadaaakeaaiiGacqWF8oqBdaWgaaWcbaGaem4AaSgabeaakiabg2da9maaqafabaWaaSaaaeaaieqacqGF4baEdaWgaaWcbaGaemyAaKgabeaaaOqaaiabd6gaUnaaBaaaleaacqWGRbWAaeqaaaaaaeaacqWGPbqAaeqaniabggHiLdaaaa@3A86@

Σ=∑k∑xi∈k(xi−μk)(xi−μk)T(n−K)
 MathType@MTEF@5@5@+=feaafiart1ev1aaatCvAUfKttLearuWrP9MDH5MBPbIqV92AaeXatLxBI9gBaebbnrfifHhDYfgasaacH8akY=wiFfYdH8Gipec8Eeeu0xXdbba9frFj0=OqFfea0dXdd9vqai=hGuQ8kuc9pgc9s8qqaq=dirpe0xb9q8qiLsFr0=vr0=vr0dc8meaabaqaciaacaGaaeqabaqabeGadaaakeaacqqHJoWucqGH9aqpdaaeqbqaamaaqafabaWaaSaaaeaacqGGOaakieqacqWF4baEdaWgaaWcbaGaemyAaKgabeaakiabgkHiTGGaciab+X7aTnaaBaaaleaacqWGRbWAaeqaaOGaeiykaKIaeiikaGIae8hEaG3aaSbaaSqaaiabdMgaPbqabaGccqGHsislcqGF8oqBdaWgaaWcbaGaem4AaSgabeaakiabcMcaPmaaCaaaleqabaGaemivaqfaaaGcbaGaeiikaGIaemOBa4MaeyOeI0Iaem4saSKaeiykaKcaaaWcbaGaem4zaC2aaSbaaWqaaiabdMgaPbqabaWccqGHiiIZcqWGRbWAaeqaniabggHiLdaaleaacqWGRbWAaeqaniabggHiLdaaaa@532D@

where *n *is the total number of training samples, *n*_*k *_is the number of training samples in class *k*, and *K *is the number of classes. In this study, *K *= 2.

#### QDA (Quadratic Discriminant Analysis)

QDA is a generalization of LDA in which each class has its own covariance matrix, S_*k*_. In this case, it can be shown [21] that the discriminant function for class *k *is equivalent to the following function:

δk(x)=−12log(|Σk|)−12(x−μk)TΣk−1(x−μk)+logπk
 MathType@MTEF@5@5@+=feaafiart1ev1aaatCvAUfKttLearuWrP9MDH5MBPbIqV92AaeXatLxBI9gBaebbnrfifHhDYfgasaacH8akY=wiFfYdH8Gipec8Eeeu0xXdbba9frFj0=OqFfea0dXdd9vqai=hGuQ8kuc9pgc9s8qqaq=dirpe0xb9q8qiLsFr0=vr0=vr0dc8meaabaqaciaacaGaaeqabaqabeGadaaakeaaiiGacqWF0oazdaWgaaWcbaGaem4AaSgabeaakiabcIcaOGqabiab+Hha4jabcMcaPiabg2da9iabgkHiTmaalaaabaGaeGymaedabaGaeGOmaidaaGqaciab9XgaSjab99gaVjab9DgaNnaabmaabaWaaqWaaeaacqqHJoWudaWgaaWcbaGaem4AaSgabeaaaOGaay5bSlaawIa7aaGaayjkaiaawMcaaiabgkHiTmaalaaabaGaeGymaedabaGaeGOmaidaaiabcIcaOiab+Hha4jabgkHiTiab=X7aTnaaBaaaleaacqWGRbWAaeqaaOGaeiykaKYaaWbaaSqabeaacqWGubavaaGccqqHJoWudaqhaaWcbaGaem4AaSgabaGaeyOeI0IaeGymaedaaOGaeiikaGIae4hEaGNaeyOeI0Iae8hVd02aaSbaaSqaaiabdUgaRbqabaGccqGGPaqkcqGHRaWkcqqFSbaBcqqFVbWBcqqFNbWzcqWFapaCdaWgaaWcbaGaem4AaSgabeaaaaa@6300@

The decision boundaries are therefore quadratic. Again, the parameters are estimated by applying maximum likelihood to the training data [21].

#### NB (Naive Bayes)

NB is based on the independent variable assumption: for each class, the variables in the feature vector **x **are assumed to be independent. This assumption allows the class conditional density *p*(*x*_*i*_|*k*) to be estimated separately for each variable, *x*_*i*_. In essence, NB reduces the problem of multi-dimensional density estimation to that of one-dimensional density estimation. Given a class, *k*, each variable in the feature vector **x **= (*x*_1_, *x*_2_, ..., *x*_*p*_)^*T *^is independent; so

p(x|k)=∏ipp(xi|k)
 MathType@MTEF@5@5@+=feaafiart1ev1aaatCvAUfKttLearuWrP9MDH5MBPbIqV92AaeXatLxBI9gBaebbnrfifHhDYfgasaacH8akY=wiFfYdH8Gipec8Eeeu0xXdbba9frFj0=OqFfea0dXdd9vqai=hGuQ8kuc9pgc9s8qqaq=dirpe0xb9q8qiLsFr0=vr0=vr0dc8meaabaqaciaacaGaaeqabaqabeGadaaakeaacqWGWbaCcqGGOaakieqacqWF4baEcqGG8baFcqWGRbWAcqGGPaqkcqGH9aqpdaqeWbqaaiabdchaWjabcIcaOiabdIha4naaBaaaleaacqWGPbqAaeqaaOGaeiiFaWNaem4AaSMaeiykaKcaleaacqWGPbqAaeaacqWGWbaCa0Gaey4dIunaaaa@4324@

Using Bayes Rule, we obtain

p(k|x)∝p(k)∏ipp(xi|k)
 MathType@MTEF@5@5@+=feaafiart1ev1aaatCvAUfKttLearuWrP9MDH5MBPbIqV92AaeXatLxBI9gBaebbnrfifHhDYfgasaacH8akY=wiFfYdH8Gipec8Eeeu0xXdbba9frFj0=OqFfea0dXdd9vqai=hGuQ8kuc9pgc9s8qqaq=dirpe0xb9q8qiLsFr0=vr0=vr0dc8meaabaqaciaacaGaaeqabaqabeGadaaakeaacqWGWbaCcqGGOaakcqWGRbWAcqGG8baFieqacqWF4baEcqGGPaqkcqGHDisTcqWGWbaCcqGGOaakcqWGRbWAcqGGPaqkdaqeWbqaaiabdchaWjabcIcaOiabdIha4naaBaaaleaacqWGPbqAaeqaaOGaeiiFaWNaem4AaSMaeiykaKcaleaacqWGPbqAaeaacqWGWbaCa0Gaey4dIunaaaa@4818@

where *p*(*k*) is the prior probability of class *k*, estimated as the ratio of the number of the training samples in class *k *to the total number of training samples. In this paper, we model each variable as a univariate Gaussian, so *p*(*x*_*i*_|*k*) = *N*(μik
 MathType@MTEF@5@5@+=feaafiart1ev1aaatCvAUfKttLearuWrP9MDH5MBPbIqV92AaeXatLxBI9gBaebbnrfifHhDYfgasaacH8akY=wiFfYdH8Gipec8Eeeu0xXdbba9frFj0=OqFfea0dXdd9vqai=hGuQ8kuc9pgc9s8qqaq=dirpe0xb9q8qiLsFr0=vr0=vr0dc8meaabaqaciaacaGaaeqabaqabeGadaaakeaaiiGacqWF8oqBdaqhaaWcbaGaemyAaKgabaGaem4AaSgaaaaa@3150@, σik
 MathType@MTEF@5@5@+=feaafiart1ev1aaatCvAUfKttLearuWrP9MDH5MBPbIqV92AaeXatLxBI9gBaebbnrfifHhDYfgasaacH8akY=wiFfYdH8Gipec8Eeeu0xXdbba9frFj0=OqFfea0dXdd9vqai=hGuQ8kuc9pgc9s8qqaq=dirpe0xb9q8qiLsFr0=vr0=vr0dc8meaabaqaciaacaGaaeqabaqabeGadaaakeaaiiGacqWFdpWCdaqhaaWcbaGaemyAaKgabaGaem4AaSgaaaaa@315D@), where the parameters μik
 MathType@MTEF@5@5@+=feaafiart1ev1aaatCvAUfKttLearuWrP9MDH5MBPbIqV92AaeXatLxBI9gBaebbnrfifHhDYfgasaacH8akY=wiFfYdH8Gipec8Eeeu0xXdbba9frFj0=OqFfea0dXdd9vqai=hGuQ8kuc9pgc9s8qqaq=dirpe0xb9q8qiLsFr0=vr0=vr0dc8meaabaqaciaacaGaaeqabaqabeGadaaakeaaiiGacqWF8oqBdaqhaaWcbaGaemyAaKgabaGaem4AaSgaaaaa@3150@ and σik
 MathType@MTEF@5@5@+=feaafiart1ev1aaatCvAUfKttLearuWrP9MDH5MBPbIqV92AaeXatLxBI9gBaebbnrfifHhDYfgasaacH8akY=wiFfYdH8Gipec8Eeeu0xXdbba9frFj0=OqFfea0dXdd9vqai=hGuQ8kuc9pgc9s8qqaq=dirpe0xb9q8qiLsFr0=vr0=vr0dc8meaabaqaciaacaGaaeqabaqabeGadaaakeaaiiGacqWFdpWCdaqhaaWcbaGaemyAaKgabaGaem4AaSgaaaaa@315D@ are estimated by applying maximum likelihood to the training data [21]. Note that NB has far fewer parameters to estimate than either LDA or QDA, and for this reason, it often performs surprisingly well in practise, despite the unrealistic assumption of independent variables [21].

#### KNN (K-Nearest Neighbors)

KNN is a nonparametric method, since it does not require the estimation of any parameters. Instead, to classify a test vector, KNN finds the vector's *K *nearest neighbors in the training data. If *K*_1 _is the number of these neighbors in Class 1, then *K*_1_*/K *is returned as the discriminant value. The test vector is therefore assigned to Class 1 if and only if *K*_1_*/K *> *τ*, where *τ *is the decision threshold.

A variety of different distance measures can be used with KNN to measure the nearness of one vector to another. In this paper, we use 1 - *ρ*, where *ρ *is the Pearson correlation coefficient of the two vectors. That is, if the two vectors are **x **and **y**, then

ρ=(x−x¯)T(y−y¯)(x−x¯)T(x−x¯)(y−y¯)T(y−y¯)
 MathType@MTEF@5@5@+=feaafiart1ev1aaatCvAUfKttLearuWrP9MDH5MBPbIqV92AaeXatLxBI9gBaebbnrfifHhDYfgasaacH8akY=wiFfYdH8Gipec8Eeeu0xXdbba9frFj0=OqFfea0dXdd9vqai=hGuQ8kuc9pgc9s8qqaq=dirpe0xb9q8qiLsFr0=vr0=vr0dc8meaabaqaciaacaGaaeqabaqabeGadaaakeaaiiGacqWFbpGCcqGH9aqpdaWcaaqaaiabcIcaOGqabiab+Hha4jabgkHiTiqb+Hha4zaaraGaeiykaKYaaWbaaSqabeaacqWGubavaaGccqGGOaakcqGF5bqEcqGHsislcuGF5bqEgaqeaiabcMcaPaqaamaakaaabaGaeiikaGIae4hEaGNaeyOeI0Iaf4hEaGNbaebacqGGPaqkdaahaaWcbeqaaiabdsfaubaakiabcIcaOiab+Hha4jabgkHiTiqb+Hha4zaaraGaeiykaKIaeiikaGIae4xEaKNaeyOeI0Iaf4xEaKNbaebacqGGPaqkdaahaaWcbeqaaiabdsfaubaakiabcIcaOiab+Lha5jabgkHiTiqb+Lha5zaaraGaeiykaKcaleqaaaaaaaa@55AC@

In terms of gene expression measurements, two genes are highly correlated if their expression levels tend to rise and fall together (even though their absolute expression levels may be quite different). For this reason, Pearson correlation is often used to detect coregulation among genes [2].

### Principal components analysis

Hidden dependencies and noise among experiments may confound the classification problem. In particular, experiments that are biologically different may actually be similar in terms of gene expression. Principal components analysis (PCA) helps to identify independent information in the data by transforming it to a data set of reduced dimension. The attributes of the reduced data set, called principal components, explain most of the variance in the original data and are mutually uncorrelated and orthogonal [21]. In addition, by reducing the dimension of the data, PCA reduces the number of parameters that must be estimated during supervised learning, thus permitting more efficient use of the data.

One can think of PCA as having a learning phase and a prediction phase. During learning, PCA is given a data set, from which it generates (learns) a linear transformation. This transformation maps high-dimensional vectors to low-dimensional vectors, and is applied to the given data set to reduce its dimensionality. During prediction, the transformation is applied to other data.

We used PCA to reduce the dimensionality of the gene expression data from its original 290 dimensions to *p *dimensions, for *p *= 5, 10, 15, 20, 40, 100. During learning, we gave PCA our entire data set of 22,746 genes, *i.e*., the 11,533 annotated genes and the 11,193 unannotated genes. This is possible because PCA is a form of unsupervised learning, so it uses only the gene expression measurements (which are known), and not the gene annotations (which are to be learned). This increases the effectiveness of PCA by doubling the amount of data that it uses during learning. That is, using a larger data set decreases the variance of the principal components learned by PCA, thus increasing their statistical significance and reducing the number of anomalous components.

It is worth noting that this use of PCA is different from that of many traditional applications of machine learning. This is because we apply PCA to the entire data set during learning, including the prediction data (*i.e*., the unannotated data). This is not possible in traditional applications simply because the prediction data is not known during learning. In such applications, a learning procedure is first trained and tested on one data set, and then applied to prediction data as it becomes available. This is not the situation for genome-wide expression experiments, since all the genes (and their expression levels) are known in advance, including the genes in the prediction set. PCA can therefore use both the prediction data and the training data during learning. This is a form of transductive inference [29,31], in which the prediction data is known and exploited during learning.

### PCA and classifier evaluation

After PCA is performed on the entire data set, supervised learning is performed on the annotated portion of the dimensionally-reduced data. (As described earlier, this is a form of semi-supervised learning [29,30]). The result is a set of classifiers, one for each supervised learning method. The classifiers are then applied to the unannotated portion of the dimensionally-reduced data to predict the missing annotations. Cross validation was used to estimate the accuracy of these predictions.

Before discussing our use of cross validation, we consider the simpler setting in which the annotated data is divided into two parts, training data and validation data [21]. This will clarify our handling of PCA during validation. In this setting, classifier evaluation proceeds as follows. First, PCA is applied to the entire data set (training, validation and prediction data) to produce a dimensionally-reduced data set. Then, a supervised learner uses the (dimensionally-reduced) training data to produce a classifier. Finally, the accuracy of the classifier is estimated using the (dimensionally-reduced) validation data. Note that this process treats the validation and prediction data equally. That is, they are *both *used during unsupervised learning, and *neither *is used during supervised learning. In this way, the validation data is representative of the prediction data, as it should be. Also note that PCA is now effectively a preprocessing phase prior to supervised learning.

Because PCA is applied to the entire data set, this validation process estimates the accuracy of the classifier on prediction data that is known and used during learning. In particular, the estimate does *not *apply to new prediction data that might arrive in the future (*e.g*., if new genes were discovered). In fact, it would likely be an overestimate of classifier accuracy on such data. However, this is not an issue in our application, since most if not all of the genes in *Arabidopsis *are already known. Moreover, even if some new genes were to be discovered in the future, we could simply add them to our prediction data and retrain the classifier on the enlarged data set.

The above ideas are easily extended to cross validation. First, PCA is applied to the entire data set. Then, a supervised learner uses the annotated portion of the dimensionally-reduced data to produce a classifier. Finally, this classifier is evaluated by cross validation in the normal way, as described below. Note that this approach has the added computational advantage that PCA is applied only once, to the entire data set, and not over-and-over again during the many training phases of cross validation. The discussions below assume that the entire data set has been preprocessed using PCA, so that all references to data refer to the dimensionally-reduced data. Also, all references to generalization performance refer to the accuracy of the classifier on the given set of prediction data.

### Cross validation

We used 20-fold cross-validation to assess the generalization performance of each classifier as well as to estimate the precision of its predictions. We randomly divided the annotated data into 20 non-overlapping, equal-sized parts, called folds. The classifier was trained on 19 of these folds, and tested on the remaining fold; *i.e*., the trained classifier was used to generate a discriminant value for each gene in the remaining fold. This was done in all 20 possible ways, using a different testing fold each time. In this way, a discriminant value, *dv*, was generated for every gene in the training set. Each gene in the training set was then predicted to be positive (*i.e*., to respond to stress) if and only if *dv *> *τ*, where *τ *is a decision threshold. From these predictions, true and false positives were computed, from which a point on the ROC_50 _curve was plotted. Using a large number of different decision thresholds, we plotted a large number of points on the ROC_50 _curve, effectively generating the entire curve. The area under this curve is the ROC_50 _score. To get an idea of how stable the estimated performance of the classifier is, we repeated the entire cross-validation and curve-generation procedure 10 times, each time using a different, random, 20-fold split of the training data.

The above procedure was applied to all the basic classifiers, but assessing the combined classifier involved an additional subtlety. Recall that the combined classifier is a linear combination of the basic classifiers, where the weight given to a basic classifier is proportional to its estimated ROC_50 _score. The subtlety is in computing that score. A naive approach would be to simply use the above procedure to compute a ROC_50 _score for each basic classifier. However, this would mean that during cross validation, 19 of the 20 folds are used to train the basic classifiers, while the 20^*th *^fold is used to compute the ROC_50 _scores. The result is that *all 20 folds *are involved in computing the weights. Thus, all 20 folds are involved in constructing (*i.e*., training) the combined classifier, so no folds are left for testing it. If cross validation were used anyway to assess the combined classifier, it would amount to using training data as testing data, and the results would tend to overestimate the classifier's performance.

As described earlier, we surmount this problem by using two sets of validation data. Loosely speaking, 18 of the 20 folds are used to train the basic classifiers, a 19^*th *^fold is used to compute their ROC_50 _scores, and the 20^*th *^fold is used to test the combined classifier. This results in what might be called *nested *cross validation. To start, the training data are divided randomly into 20 folds. Picking one of these as a testing fold, the other 19 are used to train the combined classifier. This in turn involves 19-fold cross validation to train and test the basic classifiers (and compute their ROC_50 _scores). Thus, each time the combined classifier is trained once, the basic classifiers are trained 19 times. Since the combined classifier is trained 20 times, each basic classifier is trained a total of 20 × 19 = 380 times. A similar form of nested cross validation is involved in Stacking [21].

### Predicting gene function and estimating precision

To predict which genes respond to stress, we first train a combined classifier using the 11,553 annotated genes in the training data. The classifier is then applied to the 11,193 unannotated genes in the prediction data. After this step, each annotated gene has a discriminant value, *dv*. The unannotated genes are then sorted in descending order by discriminant value, as illustrated in Table 1. To make actual predictions, a gene in the sorted list is chosen as a decision point. This gene and every gene above it in the sorted list are then predicted to respond to stress. In other words, suppose *dv *is the discriminant value of the chosen gene. An unannotated gene is then predicted to respond to stress if and only if its discriminant value is at least *dv*. The fraction of these predictions that are true is the *precision *of the predictions. We estimate this precision using the training data. Recall that each gene in the training set has a discriminant value assigned to it during cross validation. We also know which of these genes respond to stress. To estimate the precision of our predictions, we look at those genes in the training set whose discriminant value is at least *dv*. The fraction of them that respond to stress is an estimate of precision.

Using this idea we actually get ten precision estimates, not one. This is because we do cross validation ten times, using ten different random splits of the data. The result is that each gene in the training set receives ten discriminant values, and for each one we get a different precision estimate. We could simply use the average of these ten precision estimates; however, to reduce the variance of the estimate, we use a weighted average. Specifically, let us number the cross validation runs from *i *= 1, …, 10. Then, given a discriminant value, *dv*, let *PP*_*i *_be the number of genes in the training set whose discriminant values is at least *dv *in the *i*^*th *^run of cross validation. (These are the predicted positives.) Let *TP*_*i *_be the number of these genes that respond to stress (the true positives). Using only this cross validation run, the estimated precision would be *TP*_*i*_*/PP*_*i*_. One problem with this estimate is that if *dv *is high, then *PP*_*i *_(and hence *TP*_*i*_) could be 0, so the precision estimate would be undefined, something we observed frequently in practice. More generally, if *PP*_*i *_(and hence *TP*_*i*_) is low, then the precision estimate will have high variance, since it is supported by very little data. To circumvent these problems, we estimate the precision using the formula

precision=∑iTPi∑iPPi=∑iwi×TPiPPi
 MathType@MTEF@5@5@+=feaafiart1ev1aaatCvAUfKttLearuWrP9MDH5MBPbIqV92AaeXatLxBI9gBaebbnrfifHhDYfgasaacH8akY=wiFfYdH8Gipec8Eeeu0xXdbba9frFj0=OqFfea0dXdd9vqai=hGuQ8kuc9pgc9s8qqaq=dirpe0xb9q8qiLsFr0=vr0=vr0dc8meaabaqaciaacaGaaeqabaqabeGadaaakeaacqWGWbaCcqWGYbGCcqWGLbqzcqWGJbWycqWGPbqAcqWGZbWCcqWGPbqAcqWGVbWBcqWGUbGBcqGH9aqpdaWcaaqaamaaqababaGaeeivaqLaeeiuaa1aaSbaaSqaaiabdMgaPbqabaaabaGaemyAaKgabeqdcqGHris5aaGcbaWaaabeaeaacqqGqbaucqqGqbaudaWgaaWcbaGaemyAaKgabeaaaeaacqWGPbqAaeqaniabggHiLdaaaOGaeyypa0ZaaabuaeaacqWG3bWDdaWgaaWcbaGaemyAaKgabeaakiabgEna0oaalaaabaGaeeivaqLaeeiuaa1aaSbaaSqaaiabdMgaPbqabaaakeaacqqGqbaucqqGqbaudaWgaaWcbaGaemyAaKgabeaaaaaabaGaemyAaKgabeqdcqGHris5aaaa@59BB@

where wi=PPi/∑jNPPj
 MathType@MTEF@5@5@+=feaafiart1ev1aaatCvAUfKttLearuWrP9MDH5MBPbIqV92AaeXatLxBI9gBaebbnrfifHhDYfgasaacH8akY=wiFfYdH8Gipec8Eeeu0xXdbba9frFj0=OqFfea0dXdd9vqai=hGuQ8kuc9pgc9s8qqaq=dirpe0xb9q8qiLsFr0=vr0=vr0dc8meaabaqaciaacaGaaeqabaqabeGadaaakeaacqWG3bWDdaWgaaWcbaGaemyAaKgabeaakiabg2da9iabbcfaqjabbcfaqnaaBaaaleaacqWGPbqAaeqaaOGaei4la8YaaabmaeaacqqGqbaucqqGqbaudaWgaaWcbaGaemOAaOgabeaaaeaacqWGQbGAaeaacqWGobGta0GaeyyeIuoaaaa@3DCF@. The right-hand formula is a weighted average of individual precision estimates, *TP*_*i*_*/PP*_*i*_. It gives more weight to precision estimates that are based on more data, *i.e*., for which *PP*_*i *_is higher. In addition, by using the left-hand formula, we rarely end up dividing by zero, since the denominator is a sum of (random) non-negative numbers; *i.e*., Σ_*i*_*PP*_*i *_is much less likely to be zero than is any individual *PP*_*i*_.

### Biological experiments

Wild type and homozygous mutant seeds were plated on 0.5X MS media. They were stratified for 3 days and then germinated at 25C for 7 days. The abiotic temperature stresses consisted of 7 days exposure to either 30C, 14C or 4C. Anthocyanin levels were quantified as a measure of plant stress response. Anthocyanin was extracted using methanol-HCl [43]. In order to measure response to salt stress, plants were germinated for 3 days on 0.5X MS media and then transferred to medium containing 50 mM NaCl or to control plates. New root growth was measured 7 days after the transfer.

## Competing interests

The author(s) declares that there are no competing interests.

## Authors' contributions

HL and AJB did the machine learning, with HL doing the actual programming. HL developed the idea of using ROC_50 _scores to combine classifiers. RC performed the gene knockout experiments under the supervision of NJP. HL and AJB wrote the bioinformatics sections of the manuscript, with HL providing the first draft. RC wrote the biological sections. All authors read and approved the final manuscript.
